# Seasonal Variation in the Skin Transcriptome of Common Bottlenose Dolphins (*Tursiops truncatus*) from the Northern Gulf of Mexico

**DOI:** 10.1371/journal.pone.0130934

**Published:** 2015-06-25

**Authors:** Frances M. Van Dolah, Marion G. Neely, Lauren E. McGeorge, Brian C. Balmer, Gina M. Ylitalo, Eric S. Zolman, Todd Speakman, Carrie Sinclair, Nicholas M. Kellar, Patricia E. Rosel, Keith D. Mullin, Lori H. Schwacke

**Affiliations:** 1 National Centers for Coastal Ocean Science, National Ocean Service, National Oceanic and Atmospheric Administration, Charleston, South Carolina, United States of America; 2 Northwest Fisheries Science Center, National Marine Fisheries Service, National Oceanic and Atmospheric Administration, Seattle, Washington, United States of America; 3 Southeast Fisheries Science Center, National Marine Fisheries Service, National Oceanic and Atmospheric Administration, Pascagoula, Mississippi, United States of America; 4 Southwest Fisheries Science Center, National Marine Fisheries Service, National Oceanic and Atmospheric Administration, La Jolla, California, United States of America; 5 Southeast Fisheries Science Center, National Marine Fisheries Service, National Oceanic and Atmospheric Administration, Lafayette, Louisiana, United States of America; Sonoma State University, UNITED STATES

## Abstract

As long-lived predators that integrate exposures across multiple trophic levels, cetaceans are recognized as sentinels for the health of marine ecosystems. Their utility as sentinels requires the establishment of baseline health parameters. Because cetaceans are protected, measurements obtained with minimal disruption to free ranging animals are highly desirable. In this study we investigated the utility of skin gene expression profiling to monitor health and contaminant exposure in common bottlenose dolphins (*Tursiops truncatus*). Remote integument biopsies were collected in the northern Gulf of Mexico prior to the *Deepwater Horizon* oil spill (May 2010) and during summer and winter for two years following oil contamination (2010-2011). A bottlenose dolphin microarray was used to characterize the skin transcriptomes of 94 individuals from three populations: Barataria Bay, Louisiana, Chandeleur Sound, Louisiana, and Mississippi Sound, Mississippi/Alabama. Skin transcriptomes did not differ significantly between populations. In contrast, season had a profound effect on gene expression, with nearly one-third of all genes on the array differing in expression between winter and the warmer seasons (moderated T-test; p<0.01, fold-change≥1.5). Persistent organic pollutants (POPs) in blubber changed concurrently, reaching >two-fold higher concentrations in summer compared to winter, due to a seasonal decrease in blubber thickness and loss of stored lipid. However, global gene expression did not correlate strongly with seasonally changing contaminant concentrations, most likely because the refractory, lipid-stored metabolites are not substrates for phase I or II xenobiotic detoxification pathways. Rather, processes related to cell proliferation, motility, and differentiation dominated the differences in expression in winter and the warmer seasons. More subtle differences were seen between spring and summer (1.5% of genes differentially expressed). However, two presumed oil-exposed animals from spring presented gene expression profiles more similar to the summer animals (presumed exposed) than to other spring animals. Seasonal effects have not previously been considered in studies assessing gene expression in cetaceans, but clearly must be taken into account when applying transcriptomic analyses to investigate their contaminant exposure or health status.

## Introduction

Common bottlenose dolphins (*Tursiops truncatus*) are long-lived, top predators in coastal waters where they are routinely exposed to ecosystem perturbations, including anthropogenic contaminants and natural toxins, through both direct exposure and food web magnification. They have thus been identified as sentinels for the health of coastal marine ecosystems, and standard methods for temporarily capturing dolphins to assess their health and contaminant exposure have been developed and widely applied [1–4]. The utility of common bottlenose dolphins (hereafter referred to as bottlenose dolphins) as sentinels has been demonstrated through capture-release health assessments that established previously unknown transfer of PCBs through a coastal food web, culminating in dolphin health endpoints including hypothyroidism, anemia, and immunosuppression [4]. Similarly, dolphin health assessments following the explosion of the *Deepwater Horizon* drilling platform in the Gulf of Mexico and subsequent oil spill revealed an increased prevalence of lung disease and adrenal insufficiency, consistent with exposure to petroleum hydrocarbons [5]. However, the significant cost, logistical complexity, and danger of conducting capture-release studies makes the development of health indicators that can be collected from free ranging animals highly desirable, both to augment data from capture-release studies, and to obtain data from times, locations, or species not amenable to capture-release protocols.

Remote integument biopsies have been used extensively to study blubber contaminant levels in cetaceans, including bottlenose dolphins [6], since blubber is the main compartment for lipid storage. The expression level of the xenobiotic detoxification enzyme, cytochrome P450 1A1 (Cyp1A1) protein in integument biopsies has been shown to correlate with organic contaminant exposure in bottlenose dolphins [7,8] and sperm whales *(Physeter macrocephalus)* [9], and has been proposed as a biomarker for contaminant exposure. Additionally, qPCR measurement of transcript abundance of selected nuclear receptors in blubber and skin involved in xenobiotic detoxification and immune function shows correlation to persistent organic contaminant levels in harbor seals (*Phoca vitulina*) [10] and killer whales (*Orcinus orca*) [11]. Thus the transcript abundance of this suite of genes has been proposed as a suitable suite of biomarkers of contaminant exposure in cetaceans.

Since genes function in networks of coordinated pathways, the measurement of global gene expression has the potential to more precisely define the health and physiological status of cetaceans from integument biopsies. Microarray analysis of blood in bottlenose dolphins demonstrated global gene expression patterns that differed between populations from different estuaries in the southeast US and Gulf of Mexico [12]. Yet, blood transcriptomes were predictive of PCB contaminant concentrations in blubber, independent of sampling location [13]. Transcriptome studies in human skin are widely used to understand disease processes and immune functions related to wound healing and pharmaceutical effects [14,15]. In the current study, we therefore investigate the utility of skin gene expression profiling to monitor the physiology, health, and contaminant exposure in free ranging bottlenose dolphins.

The skin is a complex organ that provides many functions that may be informative of the health status of an organism. In mammals, the primary role of skin is to provide a physical barrier against the environment. The skin of cetaceans differs from that of terrestrial mammals in that it lacks hair follicles or sweat glands. Dolphin epidermis is 1–2 mm thick, 20 times thicker than that of terrestrial mammals [16]. Both a high rate of desquamation and a high rate of mitosis occur as a consequence of constant exposure to water friction [17]. The external-most *stratum externum (stratum corneum)* is not fully cornified as in terrestrial mammals, but rather is parakeratinized, consisting of flattened cells that retain their nuclei and organelles, as well as extensive intracellular keratin fibers and intracellular lipid droplets [18]. Rather than keratinocytes, which make up the majority of cells in terrestrial mammal epidermis, the dominant cell type in cetacean epidermis is the lipokeratinocyte. Lipokeratinocytes produce both keratin and lipid droplets, and contribute to the mechanical strength, buoyancy, and insulation of cetacean skin [19]. Lipokeratinocytes also secrete lamellar bodies, intercellular lipid bodies containing glycoconjugates that may aid in cell adhesion in the *stratum externum*, as well as streamlining of cetacean skin.

In addition to its role as a physical barrier, the skin is also an immune organ that contributes to both the innate and adaptive immune systems. In humans, keratinocytes produce a number of cytokines, including IL1a, IL-2, IL-6, TGFb1, TNF-a, and INF-g, as well as many of the components of the complement system [20]. Dolphin lipokeratinocytes are less well-studied, but produce β-defensin-2 and -3, which are induced by pro-inflammatory cytokines and may serve as a non-specific defense against bacteria, fungi, and algae [21]. It is currently unknown what other cytokines or complement components may be produced by lipokeratinocytes. Cells in the *stratum spinosum* produce antimicrobial compounds including lysozyme, which becomes more concentrated and found in intercellular spaces once these cells migrate into the *stratum corneum* [21]. In addition, major histocompatibility (MHC) II antigen-positive, Langerhans-like lymphocytes concentrated at the dermal-epidermal interface indicate the presence of the adaptive immune system in dolphin skin [22]. Langerhans cells, once activated, can migrate to the lymph nodes and present phagocytized antigens to activate the adaptive immune system. An extensive network of dermal papillae carries lymph and blood vessels, as well as nerve bundles deep into the epidermis, providing access to the circulating immune system and responses to the central nervous system. Skin is also considered a steroidogenic organ because it locally synthesizes and metabolizes various steroid hormones and expresses their receptors [23], and these may play a role in epidermal growth and differentiation.

Finally, the skin expresses xenobiotic pathways that serve to detoxify chemicals that pass through the *stratum corneum*, including the aryl hydrocarbon receptor (AhR) and its target, Cyp1A1. Cyp1A1 protein expression measured by immunohistochemistry is found primarily in endothelial cells of the arterioles and capillaries of the middle and deep blubber layers in dolphins [8]. However, Cyp1A1 mRNA is also expressed in the epithelium as assessed by qPCR (J. Stegeman, pers. comm.). In the killer whale both skin and blubber express AhR, as well as other xenobiotic-responsive genes including the thyroid receptor (TR), estrogen receptor (ER), interleukin 10 (IL10), and metallothionein (MT1), and their mRNA expression levels correlate with PCB levels [11].

The current study examines the skin transcriptomes from bottlenose dolphins in the northern Gulf of Mexico prior to and following exposure to crude oil resulting from the explosion of the *Deepwater Horizon* (DWH) drilling platform on April 20, 2010. An estimated 210 million gallons of oil were released into the northern Gulf of Mexico, resulting in oiling of coastal ecosystems from western Louisiana to the Florida panhandle. During the ensuing months, dolphins resident to coastal bays in the northern Gulf were observed swimming through surface oil [5]. In addition to exposure through dermal contact, dolphins were likely exposed through inhalation of volatile compounds at the air-water interface, ingestion of contaminated prey, and incidental ingestion from water or sediments while feeding. The current study utilizes a bottlenose dolphin-specific microarray to examine the skin gene expression in dolphins from three bays and sounds in the northern Gulf that received significant oiling: Barataria Bay, Louisiana, Chandeleur Sound, Louisiana, and Mississippi Sound, Mississippi, in 2010, before and after oil reached these areas, and over the subsequent year.

## Methods

### Samples

**Fig 1:**
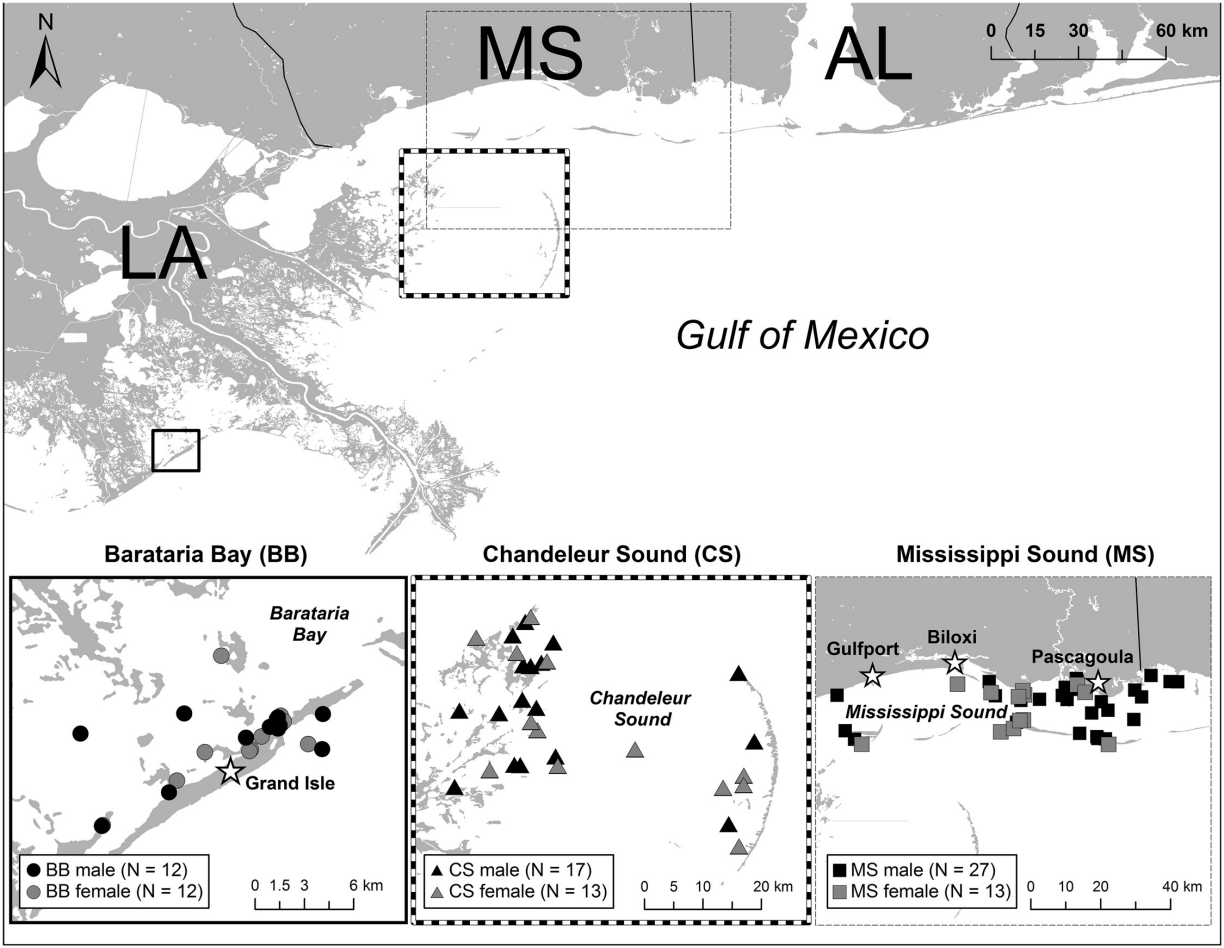
Location of biopsy samples collected from Barataria Bay (BB), Chandeleur Sound (CS), and Mississippi Sound (MS), in the northern Gulf of Mexico.

Remote biopsy samples were collected from free ranging bottlenose dolphins in three locations in the northern Gulf of Mexico, including Barataria Bay and Mississippi Sound, in May 2010, before the oil was observed on the coast, and Chandeleur Sound in late May after initial oiling. Dolphins in all three locations were sampled again in August-September 2010 after oil was present, 5–6 months later in December 2010 –February 2011, and finally the following year in August 2011 and December 2011 –February 2012 (Fig 1 and Table 1). A detailed list of individuals included in this study and their locations can be found in S1 Table. All animals sampled were subadults or adults (>10 years) as only large animals were targeted. None of the samples included in the study were repeat samples from the same individuals.

**Table 1:** Samples included in microarray analysis.

Location	Dates Collected	Number
Barataria Bay	May 10–13, 2010*	9
	Aug 14–18, 2010	4
	Jan 10-Feb 11 2011	3
	Feb 20–25, 2012	8
Chandeleur Sound	May 24–28, 2010**	2
	Sept 15–18, 2010	5
	Aug 20–24, 2011	11
	Jan 20–27, 2012	12
Mississippi Sound	May 8–17, 2010*	4
	Aug 28-Sept 16, 2010	5
	Feb 8, 2011	2
	Aug 13–29, 2011	13
	Dec 1–20, 2011	16
Total		94

*pre oil-exposure

** possible exposure; all others post-exposure

Remote biopsy samples were collected under the authority of Marine Mammal Protection Act Permit No. 779–1633, issued to the National Marine Fisheries Service (NMFS) Southeast Fisheries Science Center by the NMFS Office of Protected Species, for marine mammal research in the Gulf of Mexico, western North Atlantic and Caribbean Sea, including biopsy sampling. Institutional Animal Care and Use Committee (IACUC) approval was not required for Permit No. 779–1633. However, these biopsy sampling protocols used were approved by the NMFS Atlantic IACUC for a subsequent SEFSC permit (No. 14450). A study of the behavioral response of bottlenose dolphins to biopsy sampling using this protocol [24] demonstrated no response to minimal short-lived startle responses in 89.8% of bottlenose dolphins (acceleration or immediate dive), with only 1.7% responding strongly (breaches, tail slapping). Biopsy darts were projected using either a custom-modified 0.22-cal rifle or a commercially available Barnett Panzer V crossbow as described in [25]. Darts had an attached a solid foam float (allowing the dart to float and be retrieved after firing) and were usually recovered from the water in 60 seconds or less.

Biopsy samples were collected from the dorsal flanks of targeted dolphins typically from a ‘window’ below the dorsal fin stretching approximately 50 cm long by 20 cm high in order to minimize potential regional variation in skin gene expression. To our knowledge, no studies of variation in gene expression according to body location have been carried out. However, previous studies have shown variation in blubber fatty acid composition and contaminant concentrations according to sampling location on the animal, but found consistency within the dorsolateral region [26, 27]. Biopsies consisted of a cylindrical-shaped core of skin and blubber measuring approximately 10 mm by 15–20 mm and typically weighing between 0.5 to 1.0 g; samples were consistently judged to be full depth—extending from the epidermis to the blubbler/muscle interface. Following dart retrieval the sample was removed from the sampling tip with sterile forceps and scalpel. A subsample consisting of one quarter of the skin and blubber was taken for transcriptomics and placed in a 2.5 mL cryovial and snap frozen, then stored in a liquid N_2_ vapor shipper until transfer to the laboratory. In the laboratory, samples were stored under chain of custody at -80°C until processing.

For sex determination, genomic DNA was extracted from a subsample of the biopsied skin following Rosel et al. [28] and sex was determined using a multiplex PCR reaction which simultaneously targets the ZFX and SRY genes [29]. Pregnancy status of females was determined from concentration of progesterone in blubber [30]. Blubber cortisol was extracted and measured using previously developed methods used for blubber steroid isolation [31].

### RNA extraction

Samples were processed in two tiers, at the end of year 1 (May 2010-Feb 2011) and at the end of the second year (August 2011-Feb 2012). Within a tier, all samples were randomized prior to RNA extraction. Preliminary RNA extraction trials from different integument depths did not recover sufficient RNA from blubber at any depth. Therefore, for this study approximately 0.05–0.15 g of skin was dissected from frozen integument using a scalpel, on dry ice, just medial to a white line demarking the fibrous layer of the dermis, and further cut into pieces ≤ 0.2 cm thick. The dissected sample was transferred to 1.5 ml of RNA*later* ICE (Ambion) prechilled to -80°C and incubated for at least 16 hours at -20°C to transition the tissue from -80°C to -20°C prior to processing. The sample was then removed from the RNA*later* ICE and placed in a 2.0 ml microcentrifuge tube containing 1ml Qiazol (Qiagen) and a 5 mm stainless steel bead, and homogenized 3x using a Qiagen Tissuelyser at 20 Hz for 3 min. The sample was cooled on ice for 30 sec between sets. The homogenate was transferred to a new 2.0ml microcentrifuge tube and incubated on the benchtop at room temperature for 5 minutes before adding 200μl of chloroform. The tube was shaken vigorously for 15 seconds then incubated on the benchtop at room temperature for 3 minutes. After 15 min of centrifugation at 12,000 x g at 4°C, 500 μl of the upper aqueous phase was transferred to a new 1.5 ml microcentrifuge tube containing 500 μl of 70% ethanol, mixed by pipetting, and then immediately transferred to a Qiagen RNeasy spin column. The protocol for Purification of Total RNA using the RNeasy Lipid Tissue Mini Kit with on-column DNase digestion (Qiagen RNeasy Lipid Tissue Handbook 02/2009) was followed exactly from this point on with a 60 μl final elution in RNase-free water. The RNA quantity was determined using a Nanodrop ND-1000 spectrophotometer and quality was assessed using an Agilent 2100 Bioanalyzer. Only samples with an RNA integrity number (RIN) of ≥ 7.0 were used in the study.

### Microarray

The bottlenose dolphin microarray used in this work is a custom 44K oligonucleotide array designed using Agilent’s E-array platform (Amidad #028889) [13]. Details on the array are available in the National Center for Biotechnology Information (NCBI) Gene Expression Omnibus (Platform GPL17696). The array included 24,418 unigene sequences obtained from the publicly available NCBI expressed sequence collection (dbEST), short read archive (SRA), and nucleotide database (nr), which were quality trimmed, and assembled into contigs. These sequences originated from cDNA libraries for dolphin peripheral blood leukocytes (PBL), liver, kidney, spleen, muscle and skin, and individual cloned genes from lung. The sequence set was annotated using Blast2GO [30] to obtain BLASTx homology (expect value cutoff E≤10^-3^) gene ontology (GO) terms, and enzyme codes to determine metabolic pathways using the Kyoto Encyclopedia of Genes and Genomes (KEGG) database. Sixty-nucleotide probes designed by eArray (Agilent) were printed in a 4x44K format. Of the 24,418 sequences represented on the microarray, 7281 were fully annotated.

### RNA Labeling and Microarray Hybridization

Within each sampling tier (as defined above), RNA samples were randomized prior to labeling, then randomized again prior to hybridization, to avoid any potential batch effects associated with either the labeling or hybridization procedures. Total RNA (50ng) was amplified and Cy 3 labeled using Agilent’s Low Input Quick Amp Labeling Kit. The Cy3 labeled cRNA was quantified using a Nanodrop ND-1000 spectrophotometer. A total of 1.65 μg of Cy3 labeled cRNA was hybridized to the microarray. The One-Color Microarray-Based Gene Expression Analysis Protocol (Version 6.5, May 2010) was followed for both the labeling and the hybridization. Hybridization was carried out at 65°C in an Agilent hybridization oven rotating at 10 rpm for 17 hours. Slides were scanned with an Agilent G2505B scanner equipped with Agilent Scan Control software, using the Extended Dynamic Range Scan Mode, 5μM scan, XDR PMT Hi 100% Lo 10%. Images were extracted using Agilent Feature Extraction version 10.7.3.1.

### Data Analysis

Microarray scan quality was assessed using Agilent’s Feature Extraction software. QC reports and text (.txt) files obtained from Agilent Feature Extraction (FE) were imported into Agilent’s GeneSpring 11.5.1 software and processed as Agilent single color arrays. Normalized signal values were generated by setting the threshold for the raw signals to 1.0, log transformation, and 75th percentile shift normalization. Between-array consistency was then assessed using GeneSpring’s quality control pipeline. Principal components analysis was performed in Genespring using default parameters. Differential expression between selected conditions was determined by volcano analysis using a moderated T-test with Benjamini-Hochberg multiple test corrected p-value of 0.01. A minimum fold-change for inclusion in the T-tests was ≥1.5. This fold-change on Agilent platform arrays has previously been found to yield good correlation with qPCR analysis [33]. Unsupervised hierarchical clustering was carried out in Genespring using Euclidean distance metric and Wards linkage rule. Gene ontology enrichment analyses were carried out in Blast2GO [32] using Fishers Exact Test with a false discovery rate (FDR) of 0.05. GO ontologies were then reduced to the most specific terms.

## Results and Discussion

### RNA Quality Obtained from Remote Biopsy Collections

Of 239 remote biopsy samples collected, 101 (42%) yielded sufficiently high quality skin RNA to use in the microarray analysis, using a minimum acceptable RNA integrity value of ≥7.0. Sampling season (i.e., ambient temperature) did not appear to contribute significantly to the variability in sample quality. Rather, inconsistency in RNA quality likely reflects the variability in the time samples remained exposed to ambient air and water during retrieval of the sampling tip, as well as time taken to process the samples once on board the vessel. Based on the results of this study, for subsequent studies we have implemented a field protocol wherein retrieved biopsies are subsampled for gene expression analysis on an ice-cooled cutting board and placed immediately in RNA*later*. We found that this collection method results in 100% of samples yielding high quality RNA. Nonetheless, for the current study sufficient samples with high quality RNA were available to conduct gene expression analysis.

After removing suspected coastal stock animals (those collected where the Chandeleur Sound and coastal stocks may co-mingle), 94 high quality RNA samples were labeled and hybridized to a custom bottlenose dolphin oligonucleotide microarray. Samples were processed in two tiers, year one and year two following the oil spill. Within a tier, samples were randomized at the point of RNA extraction, labeling, and hybridization to minimize batch effects that can affect microarray analyses. Quality control analyses within Genespring did not indicate any bias due to the two tiers of sample processing. All data have been deposited in the Gene Expression Omnibus (GEO) as accession #GSE58471.

Of 24,418 probes on the microarray, 22,067 probes were detected in skin RNA samples in at least 10 percent of the samples. Given that the microarray probes were designed from cDNA libraries to multiple bottlenose dolphin tissues (see Methods), this degree of hybridization by skin RNA suggests that the array contains many functions expressed by multiple cell types. How many array probes are skin-specific is unknown.

### Genes in Dolphin Skin Differentially Expressed According to Sex

In order to ensure that sex-specific gene expression did not confound the analysis of dolphin skin samples, we first compared male and female gene expression patterns. Thirty-eight females and 56 males were included in the study, distributed among all three sampling sites and during all seasons (Fig 1 and S1 Table). Of the 22,067 probes detected in skin, 44 (0.20%) were detected only in females, and 41 (0.19%) were expressed only in males. In addition, among the probes detected in both males and females, 4 were differentially expressed by sex at greater than 2-fold in normalized log intensity values (moderated T-test p≤0.05). Among the annotated sex-specific genes were several that, in humans, are located on the X chromosome (S2 Table). In all further analyses we used a set of 21,978 probes consisting of only probes present in both males and females, minus 4 probes that were more than two-fold different in expression between the two sexes. Sex-specific gene expression patterns, previously reported in the blood of bottlenose dolphins [12], were shown to have an impact on the ability to use transcriptome profiles for classification.

We also investigated the effects of pregnancy status on skin gene expression in females, since bottlenose dolphin reproduction tends to be seasonally synchronized, with calves in the northern Gulf of Mexico generally born in the spring [34,35]. In the spring 2010 samples, 5 of 9 females were determined to be pregnant, as assessed by blubber progesterone levels of >40 ng/g [30], whereas only three samples, two from August and one from January, indicated pregnancy during all subsequent sampling periods. No significant differences in skin gene expression profiles were observed (moderated T-test, p<0.05) between pregnant and non-pregnant females samples during the spring. Therefore, all females were included in analyses independent of pregnancy status.

### Season Is a Significant Driver of Skin Gene Expression in Dolphins

**Fig 2:**
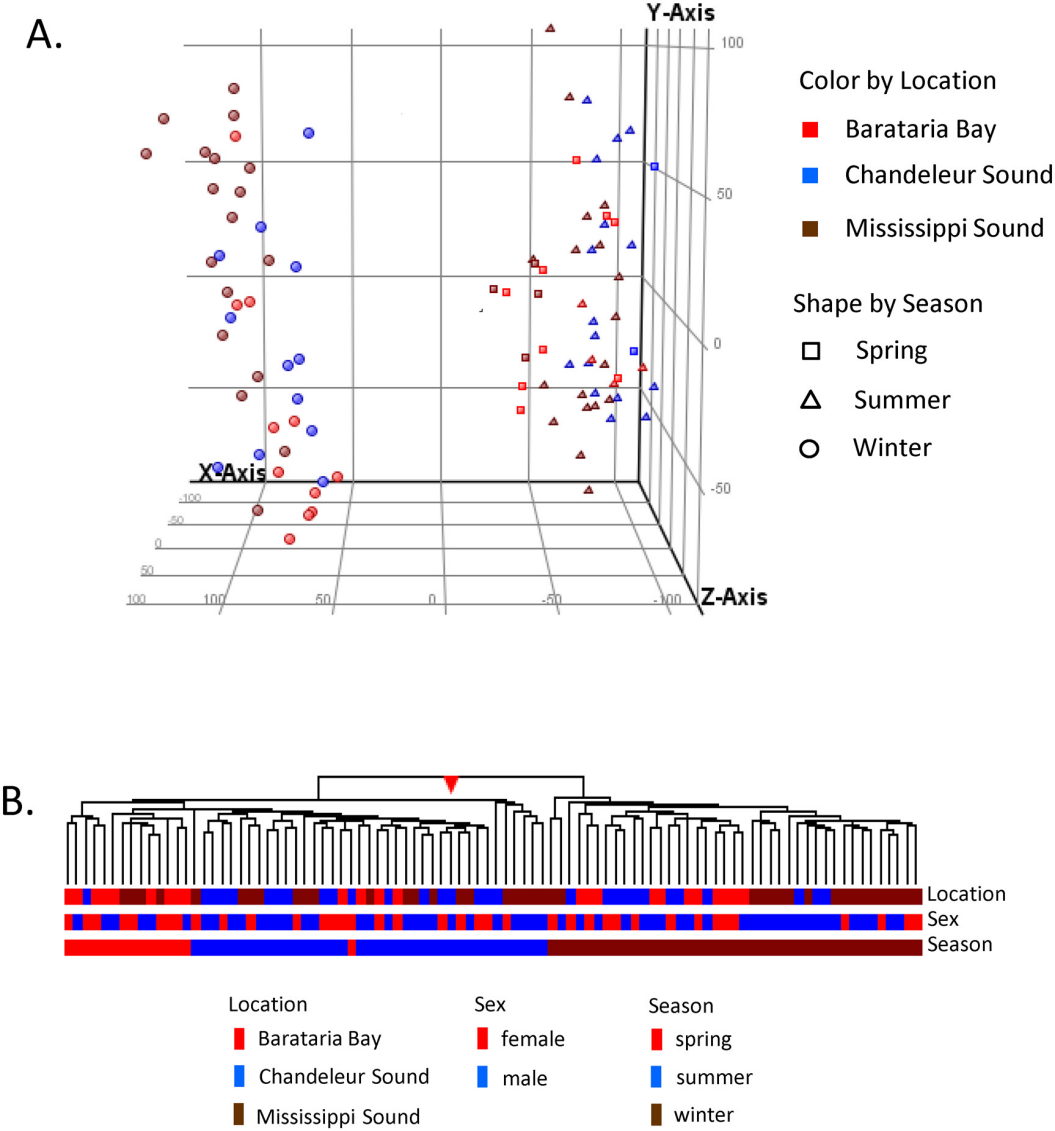
A. Principal component analysis on all 94 microarrays shows complete separation of winter samples from all others, with no apparent segregation according to location. B. Unsupervised hierarchical cluster analysis of the normalized array data similarly shows complete separation of winter samples from the warmer seasons.

Principal components analysis on all 94 samples revealed a strong effect of season on the global gene expression profiles (Fig 2a), where winter samples diverged from the other seasons along the x- axis (first principal component). This principal component accounted for 32.6% of all variation among samples. In contrast, there was no grouping of samples according to location, even though significant genetic differentiation at neutral markers has been found in adjacent bays in the Gulf of Mexico [36]. This finding differs from studies of blood gene expression in dolphins, in which samples from different estuarine populations were distinguishable by microarray [12].

Unsupervised hierarchical cluster analysis similarly showed complete stratification of gene expression profiles according to season, with winter samples clustering separately from spring and summer samples (Fig 2b). Neither sex nor location showed significant clustering. To explore the basis for this strong seasonal difference, we performed a moderated T-test to identify only those probes significantly differentially expressed between winter and other seasons. This resulted in 5443 gene probes (24.7%) significantly different in winter (p<0.01, fold change ≥1.5), relative to the other seasons (S3 Table). Gene ontology enrichment analysis (Fishers exact test, FDR 0.05) found the following GO process terms over-represented: nuclear envelope organization, nuclear migration along microfilament, protein localization to nucleus, fibroblast migration, positive regulation of endothelial cell proliferation, purine nucleobase metabolism, retrograde axon cargo transport, and protein oligomerization. Of these, 2960 genes were more highly expressed in spring/summer, while 2483 genes had higher expression in winter. The GO process terms over-represented in the set more highly expressed in summer mirror those of the whole set (S4 Table). In contrast, GO processes enriched in the gene probes more highly expressed in winter included: regulation of glucose transport, seryl-tRNA aminoacylation, and regulation of ketone metabolic process. Overall, the GO term enrichment analysis suggests seasonal differences in cell proliferation and cell migration/maturation, which likely reflect decreased metabolic rate and rate of regeneration of skin in winter as compared to warmer months. Since the epidermis lies outside of the thermal protective blubber layer in dolphins, it experiences significant changes in temperature between seasons. For example, in Sarasota Bay, FL, where water temperatures range seasonally from 11–33°, the skin surface temperature in bottlenose dolphins varied from 18.1 ± 0.4°C in winter to 30.8 ± 0.2°C in summer [37]. Although measurements of skin temperature are not available for the current study, the lowest water temperature recorded during the winter sampling was 7.8°C, while the highest temperature recorded during the summer sampling was 34.2°C.

### Skin Transcriptome Profiles in Spring vs Summer 2010

**Fig 3:**
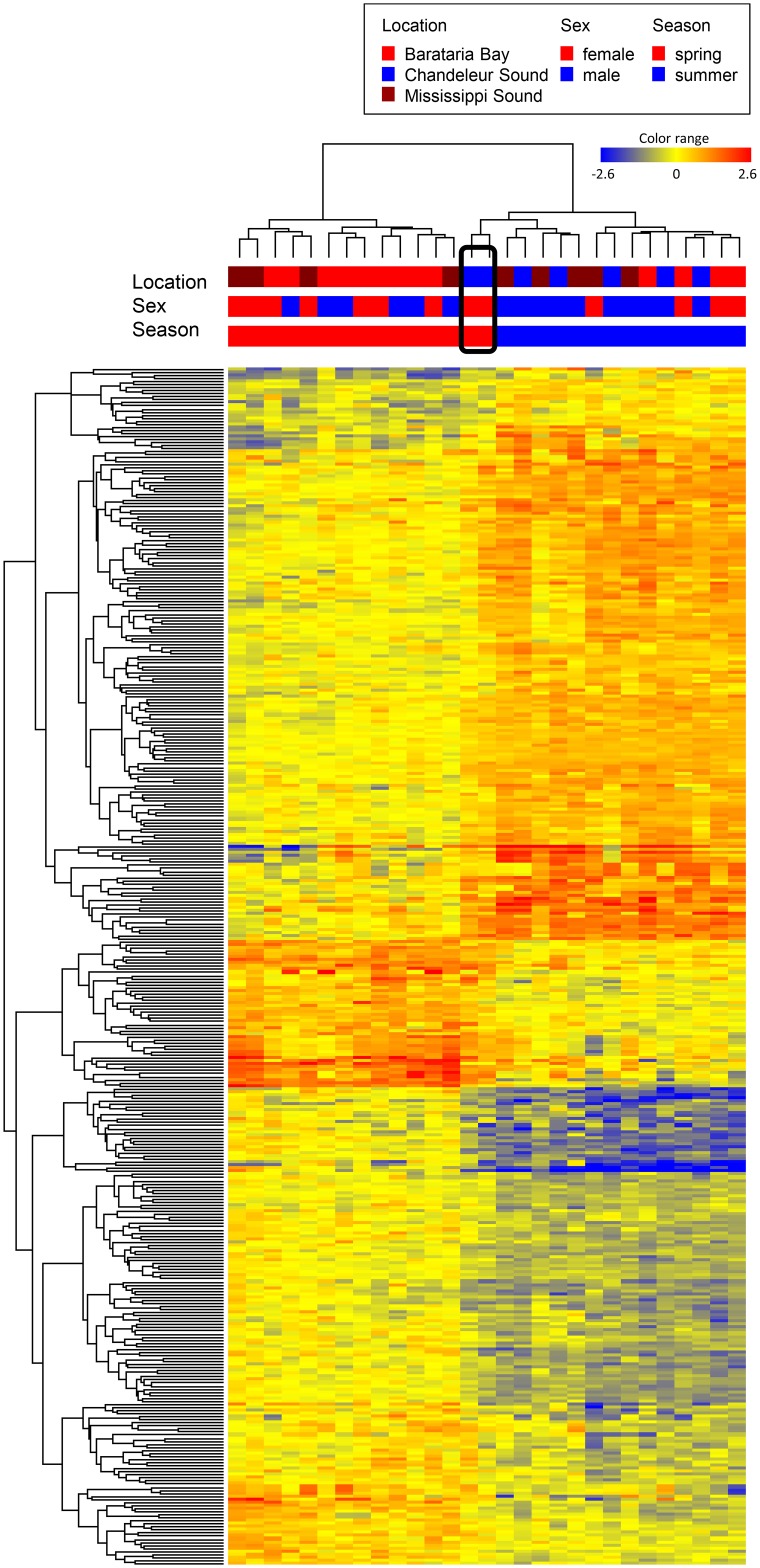
Two-dimensional hierarchical cluster analysis of 391 probes differentially expressed in 29 samples collected from spring and summer 2010 (pre- and post-oiling) (p<0.01, fold change ≥1.5). Spring Chandeleur Sound (boxed) animals cluster with the summer expression profiles. Heat map color is relative to the median intensity for each probe: red is increased, blue is decreased, log_2_ scale.

In addition to the substantial differences between winter and the warmer seasons, the hierarchical cluster also suggests some differences between spring and summer skin gene expression profiles (left hand cluster, Fig 2b). In general, the expression levels observed in spring are intermediate between those of summer and winter. In order to remove the overarching effect of winter on the analysis, we compared expression profiles only in samples collected in May (Spring, presumed pre-oil) and August-September 2010 (Summer, presumed oil exposed). Barataria Bay and Mississippi Sound were sampled in early-mid May prior to oil reaching the coast. Chandeleur Sound, however, was not sampled until May 24–28, by which date the dolphins sampled may have been exposed to oil, based on oiling maps compiled by synthetic aperture radar mapping [38], although no overt oiling was apparent on or in the vicinity of the dolphins at the time of sample collection. Additional dolphins were sampled from the same three sites in August-September, three to four months following the initial oiling in these locations. Three hundred ninety-one probes (1.8%) were differentially expressed between the spring and summer samples (moderated T-test, p<0.01, 1.5 Fold-change; S5 Table). GO process terms “somatic muscle development” and “regulation of actin filament” were enriched among this probe set. When those more highly expressed in spring (204) are considered separately, the GO process terms “muscle filament sliding” and “muscle organ development” were enriched, in addition to the GOs identified in the whole set. No significant GO enrichment was found among probes more highly expressed in summer (187). Interestingly, the two potentially oil-exposed females sampled from Chandeleur Sound on May 27 and 28 clustered with the summer animals (Fig 3, boxed). This suggests that some of the differences in their expression profiles could reflect oil exposure and not solely seasonality; however, the small sample size (n = 2) from Chandeleur Sound in May precludes any conclusive interpretation.

### Transcriptome Expression in Summer 2010 vs Summer 2011

Using the same significance criteria above (moderated T-test, p<0.01; 1.5-fold change), gene expression between summers was found to be quite stable, with only 52 gene probes (0.2%) differing in expression between pooled samples from all locations during summer 2010 and summer 2011 (S6 Table). Among these, only 16 were annotated (Expect values <1e-07) and no significant enrichment of GO processes was observed.

### Seasonal Variation in Persistent Organic Contaminants

**Fig 4:**
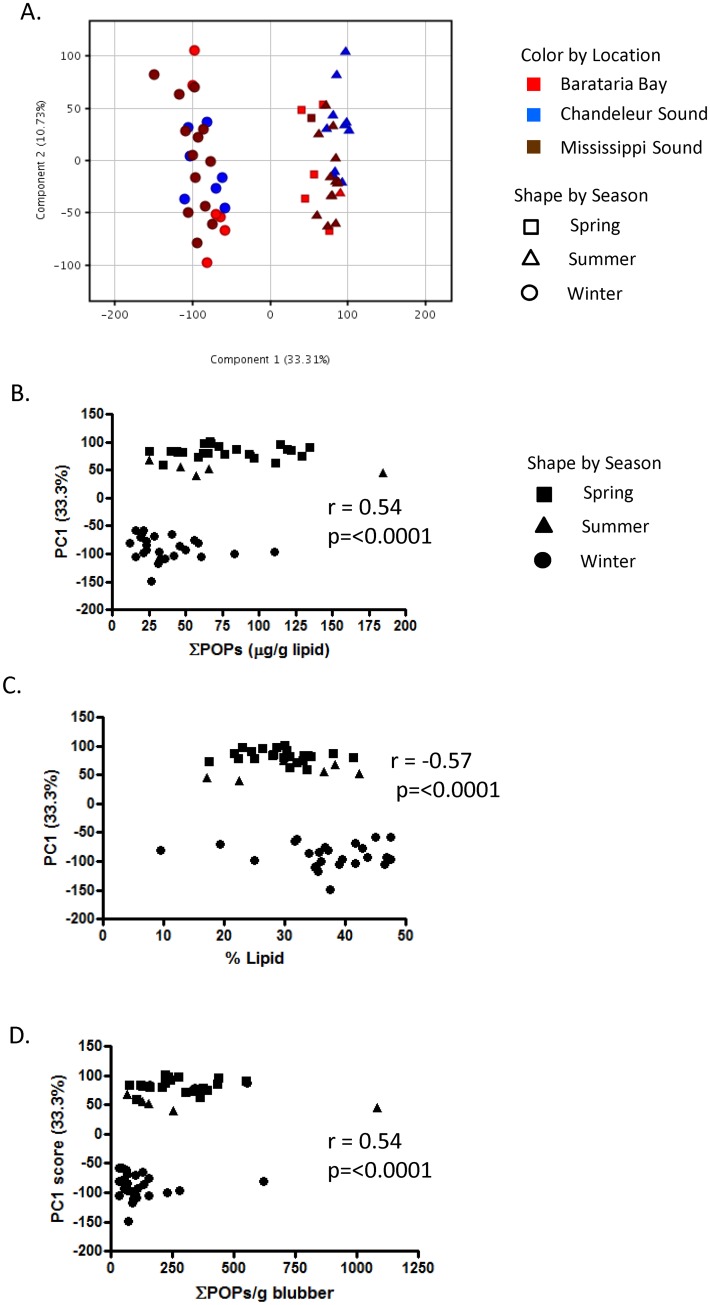
A. Principal component analysis of 56 males shows complete separation between winter samples (circles) and those from spring (squares) or summer (triangles). B. A significant positive correlation (Spearman rank) exists between PC1 sample scores and ∑POPs in blubber. C. An equivalent negative correlation exists between blubber % lipid and PC1. D. ∑POPs reported on a blubber wet weight basis has the same correlation as lipid normalized ∑POPs.

**Fig 5:**
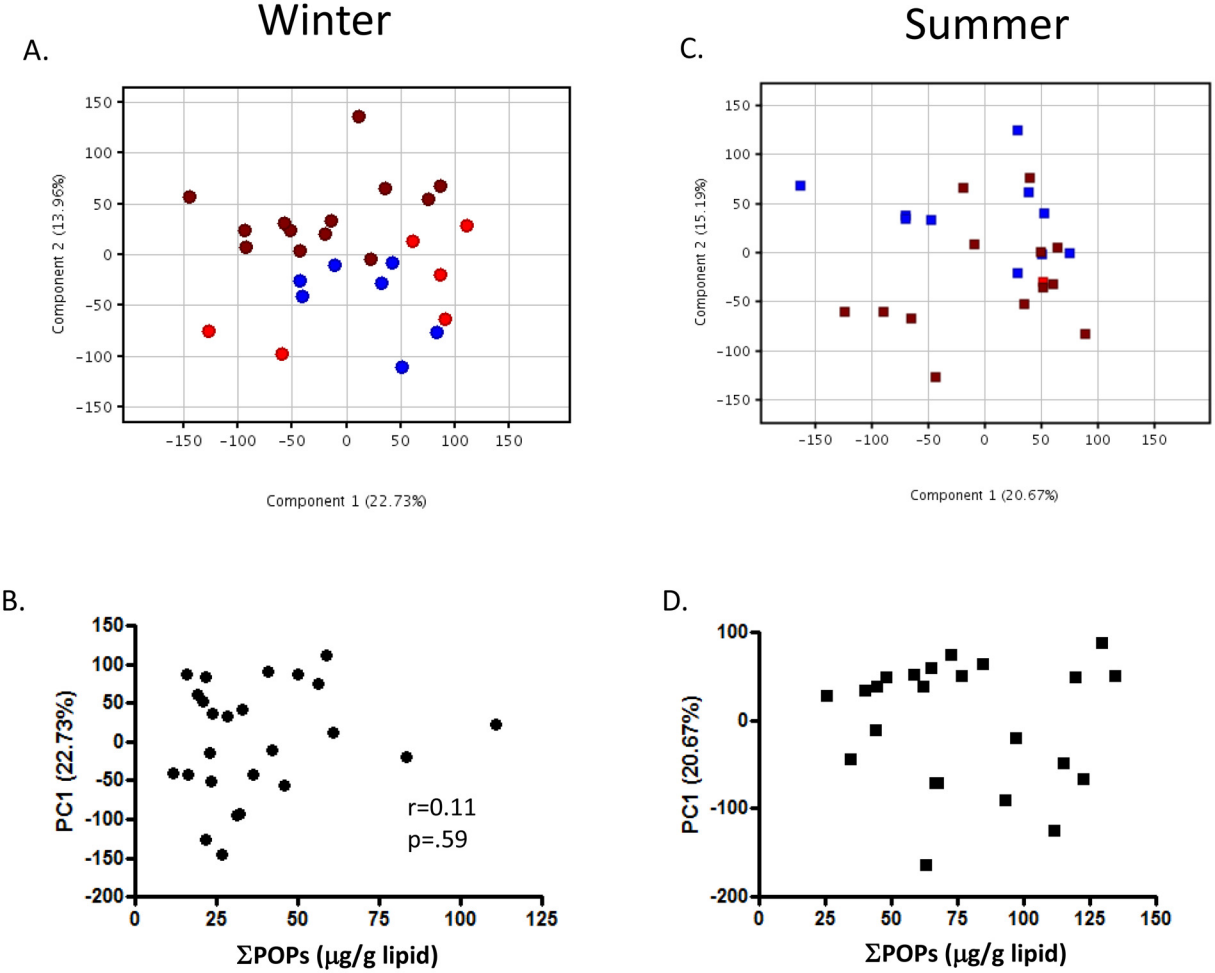
Principal components analyses on males from within a season: winter (A), summer (C). Within a season, there is no significant correlation (Spearman rank) between ∑POPs in blubber (μg/g lipid) and the PC1 scores, which are responsible for 22.7% (winter, B) and 20.6% (summer, D) of the total variance in the gene expression profiles. Symbols: red—Barataria Bay, blue—Chandeleur Sound, brown—Mississippi Sound.

Bottlenose dolphin blubber represents the lipid-rich hypodermis, and is the primary site for accumulation of lipophilic contaminants, accounting for >90% of the total body burden [36]. It is therefore often used to estimate contaminant exposure. Because females offload significant amounts of contaminants to their young through lactation [39], blubber contaminant concentrations are often monitored in males as a means to assess cumulative exposures. However, in all animals blubber undergoes seasonal changes in composition through the accumulation and mobilization of lipid stores from adipocytes. A decrease in lipid content of the blubber, due to temperature, starvation, or disease, is accompanied by a selective redistribution of a portion of these stored contaminants to the blood and other tissues [40, 41]. A recent survey of POPs (PCBs, chlordanes, dieldrin, Mirex, DDTs, BDEs, HCB, endosulfan) conducted on 175 male dolphin biopsies from the northern Gulf of Mexico [39] included all but two of the male animals in the current study. Among these animals, the ∑POPs in the blubber was significantly higher in spring/summer than in winter (S1 Table): 77.6 ± 37.5 μg/g lipid in spring/summer versus 37.3 ± 23.0 μg/g lipid in winter (t-test; p<0.0001), with all POPs classes following similar trends. Concurrently, the blubber lipid content was 20% lower in spring/summer than in winter: 29.7 ± 6.44% in spring/summer versus 36.88 ± 8.88% of the total blubber mass in winter [39]. These observations led us to investigate how much of the seasonal changes in epidermal gene expression might be attributed to changes in the contaminant burden in the deep dermal layers. PCA was used to explore these relationships in 56 male dolphins. As observed above for the entire dataset, a complete separation of gene expression profiles from winter animals and spring/summer males was found, with the first principal component accounting for 33.3% of all variance in the data (Fig 4a). ∑POPs in the blubber (μg/g lipid) had a significant correlation (r = 0.54; p<0.0001) with the first principal component (Fig 4b), which was mirrored by a negative correlation between %lipid and the first principal component (r = -0.57; p<0.0001) (Fig 4c). When reported as μg/g wet weight blubber, the relationship does not change (r = 0.54, p<0.0001). However, correlation does not infer a causative relationship between ∑POPs and global gene expression. When males from within a season were analyzed separately by PCA, 22.7% (winter) or 20.7% (summer) of the variance was explained by the first principal components; however, there was no significant correlation between PC1 (or PC2 or PC3) and ∑POPs in the blubber in either season (Fig 5). The absence of a correlation between global gene expression in the epidermis and ∑POPs in the blubber may reflect the fact that the stored POPs found in blubber, and mobilized seasonally to other tissues, consist largely of refractory metabolites that have already been processed through the phase I and phase II detoxification pathways; therefore, when mobilized these compounds do not present substrates that would elicit the induction of canonical detoxification pathways. It is also possible that the epidermis is not exposed to sufficient amounts of circulating contaminants in the blood to induce a transcriptomic response, or that the xenobiotic pathways in dolphin epidermis are not highly inducible by systemic sources.

### Response of Xenobiotic Pathway Components on the Microarray

Given the absence of a correlation between blubber contaminant levels and global gene expression in skin, we next queried specifically the expression profiles of genes involved in xenobiotic pathways, which might be responding to new chemical exposure, particularly oil associated compounds such as polycyclic aromatic hydrocarbons, known to induce the aryl hydrocarbon receptor and p540 mediated detoxification. There is currently little information on the expression of xenobiotic pathways in the dolphin epidermis. Cyp1A1 protein, the most widely studied biomarker of exposure to organic contaminants in cetaceans, was found to be expressed primarily in the endothelial cells of the arterioles and capillaries of the middle and deep blubber layers, as assessed by immunohistochemistry, but was not detected using this method in epidermal *basale*, *spinosum*, or *corneum* layers (lipokeratinocytes) or melanocytes of the epidermis [8]. However, Cyp1A1 transcripts are expressed in the epidermis as measured by qPCR (J. Stegeman, pers. comm.). In killer whales, both skin and blubber express mRNA for a number of nuclear receptors known to activate Cyps and other xenobiotic pathway genes, including the aryl hydrocarbon receptor (AhR), thyroid receptor (TR), retinoid x receptor (RxR), and glucocorticoid receptor (GR) [11]. Similarly, striped dolphins (*Stenella coeruleoalba*) express mRNAs encoding AhR, Cyp1A, Cyp2B, and ER in skin biopsies [42].

Better studied, human epidermis expresses an abundance of different phase I and phase II detoxification enzymes, including Cyps, glutathione transferases, sulfotransferases, and epoxide hydrolases [43,44,45]. Although a large variety of CYPs are found in human epidermis, a recent proteomic profiling study found their expression levels to be about 300-fold lower than found in liver, the main organ for detoxification of xenobiotics [46]. However, the presence and expression levels of Cyps in skin appear to be species dependent; for example, rodent skin expresses significantly higher levels than human skin. The dolphin microarray used in the current study contains 12 probes representing 8 Cyp family members, but does not have probes for the AhR or for AhR-dependent genes Cyp1A, Cyp1B or Cyp2B classically induced by POPs and oil-associated hydrocarbons. Cyp2E1, inducible by alcohol and benzene, is represented on the array by two probes and is reported in human skin [45], but was non-detectable on the array in dolphin skin. The Cyp3A family is represented by 5 probes with top blast hits to Cyp3A29 and Cyp3A89. Cyp3A29 is a homolog of human Cyp3A4, which is activated by PXR, a mediator of xenobiotic responses, and IFNα known to induce antiviral mechanisms and immune responses [47]. Cyp3A89 was described from the horse genome sequence; its activity remains to be fully characterized. Three Cyp4 family members present on the array, Cyp4F4, Cyp4F6, and Cyp4V2, were expressed in dolphin skin. Like other Cyp4 family members these genes are likely to be involved in the homeostasis of fatty acids and fatty acid derived inflammatory mediators [48]. Cyp26A1, involved in retinoic acid metabolism and present on the array, was non-detectable in the dolphin skin samples.

**Fig 6:**
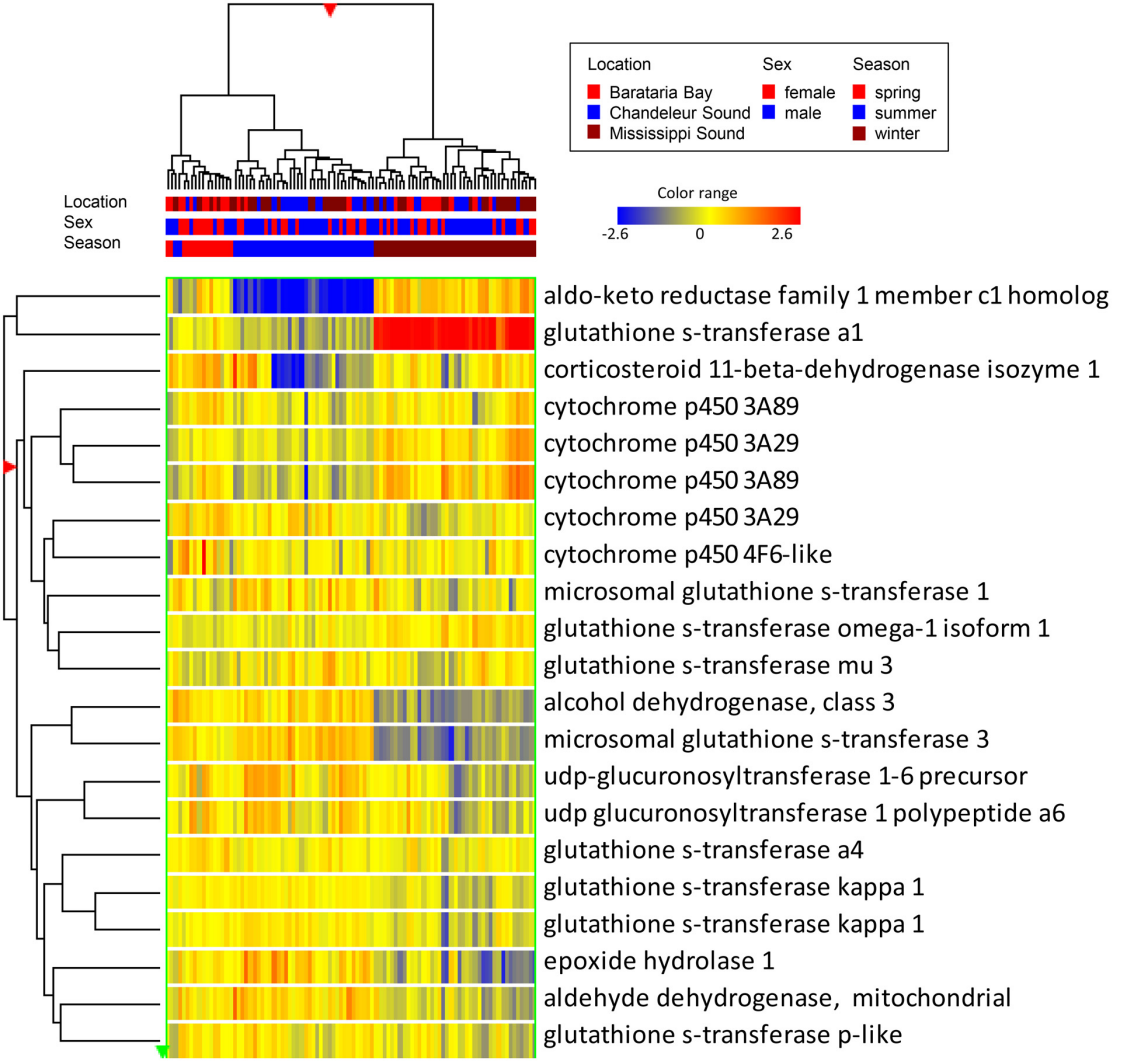
Hierarchical cluster of probes mapping to the KEGG pathway “Metabolism of xenobiotics by cytochrome p450”. Heat map color is relative to the median intensity for each probe: red is increased, blue is decreased, log_2_ scale.

We queried the relative expression of genes that mapped to KEGG pathway “Metabolism of xenobiotics by cytochrome p450”, which included the Cyp3 family and one Cyp4 family member discussed above. There was a seasonal trend in the expression of many genes in this pathway (Fig 6), with similar patterns in both males and females. Generally the Cyp3A gene probes showed increased expression in winter relative to summer, whereas Cyp4F6-like did not show seasonal variation. Because there is considerable crosstalk between the xenobiotic pathway and metabolic pathways for glucocorticoids, steroids, and cytokines, interpretation of the xenobiotic pathway response, relative to seasonal changes in stored POPs or to the oiling event, is not straightforward.

The most robust seasonal changes in this pathway were seen in the expression of aldo keto reductase family 1 member C1 (AKR1C1), which showed strong down regulation in summer, when the POPs in the blubber are most concentrated and their distribution in blood and other tissues is expected to be highest. AKR1C1(also known as dihydrodiol dehydrogenase 1) is part of an AKR superfamily that catalyzes the reduction of aldehyde and ketone groups of various substrates to their corresponding alcohols. AKR1C1 mRNA is inducible by PAHs via an ROS-dependent pathway, rather than the AhR pathway [49]; however, its downregulation in the months following oil exposure is not consistent with PAH responsive activity. AKR1C1 also acts to convert progesterone to its inactive form, 20-alpha-hydroxy progesterone; thus its downregulation in summer might be anticipated to result in increased progesterone levels. However, blubber progesterone levels in males did not differ significantly between winter and summer (winter 0.47 ± 0.37 ng/g; summer 0.42 ± 0.29 ng/g) nor did males differ greatly from non-pregnant females during summer (0.39 ± 0.15 ng/g; winter levels were variable and higher). The role of AKR1C1 in skin is not well studied. In human keratinocytes, inhibition of AKR1C1 expression is caused by UV light exposure in an ROS-dependent manner and inhibition of its expression by siRNA leads to significant reduction in cell viability [50]. To our knowledge the seasonal expression variation of AKR1C1 has not been explored in any species.

A similar decrease in expression during the summer is seen in corticosteroid 11-β-dehydrogenase isozyme 1 (HSD11B1), which primarily converts cortisone to active cortisol. Human epidermal keratinocytes synthesize cortisol and regulate its synthesis and activity in response to wounding [51]. Cortisol has growth-inhibiting and/or pro-apoptotic effects; thus a decrease in epidermal HSD11B1expression during summer may be a protective mechanism against the negative effects of cortisol on cell proliferation during a season when epithelial regeneration may be especially important. The decreased expression of HSD11B1in summer does not correspond with seasonal blubber cortisol levels, which are unchanged in males (0.83± 0.63ng/g winter; 0.80± 0.54 ng/g summer) or non-pregnant females (0.45 ± 0.28 ng/g winter; 0.45 ± 0.39 ng/g summer). Rather, it likely reflects local regulation within the epidermis. In addition to its role in glucocorticoid metabolism, corticosteroid 11-β-dehydrogenase acts as a phase I detoxification enzyme that can reduce carbonyl groups on a variety of xenobiotics [52], and thus it remains a candidate for xenobiotic activity in the epidermis.

Several members of the glutathione transferase family are represented on the array, including cytosolic GSTα1, GSTα4, GSTκ, GSTμ, GSTω, and GSTπ and microsomal GSTs 1 and 3. Glutathione transferases (GSTs) are phase II enzymes that conjugate xenobiotics and products of oxidative stress to glutathione, thereby increasing their polarity and availability for excretion. The expression patterns varied among different GST family members. Most marked was the strong upregulation of cytosolic GSTα1 in winter. GSTα1 expression is regulated by the AhR, and its substrates include polycyclic aromatic hydrocarbons. In humans exposed to dioxins, both AhR and GSTα1are upregulated in the skin [53]. However, in addition to its role in xenobiotic metabolism, GSTα1 also has glutathione peroxidase activity and is responsive to endogenous products of oxidative stress. Microsomal GST3 had the opposite pattern, with strongly decreased expression in winter. The other GSTs showed no strong seasonal changes. UDP-glucuronosyl transferases, another family of phase II enzymes that conjugate substrates to glucuronic acid, showed somewhat lower expression in winter than in summer samples.

## Summary

This study was undertaken to explore the utility of the bottlenose dolphin skin transcriptome in monitoring exposure and health impacts of environmental perturbations such as chemical spills. We found that season has a profound effect on gene expression, with nearly one-third of all genes on the array differing significantly in expression levels between winter and the warmer seasons. Gene ontology categories over-represented in the differentially expressed gene set are consistent with known seasonal changes in the physiology of dolphin skin. Although concentrations of POPs stored in blubber increased more than 2-fold in spring/summer due to the seasonal mobilization of lipid from the blubber, the observed gene expression patterns did not appear to be driven by POPs concentrations. It is currently unknown if this is because the epidermis is unresponsive to changes in the blubber, or if the POP metabolites mobilized concurrent with the loss of blubber lipid are not suitable substrates for the phase I and phase II pathways. The microarray used in the current study lacked probes to several key genes induced by and responsible for the detoxification of POPs or oil-associated hydrocarbons (e.g., AhR, Cyp1A1). The examination of xenobiotic pathway responses in this study was thus limited by incomplete pathway representation on the microarray, and complicated by crosstalk between steroid metabolism and xenobiotic pathways. Direct RNA sequencing (RNAseq) analyses, currently in progress, will retrieve critical xenobiotic pathway components known to be expressed in dolphin skin, as well as provide a deeper context for the seasonally driven physiological changes observed in the current study. Controlled exposure studies in skin biopsy samples or dolphin skin cells will help sort out xenobiotic induction of skin gene expression from that resulting from normal physiological changes. This study found no difference in global skin gene expression between adjacent bays; however, investigations need to be conducted to compare more distant regions before a clear picture of variation by location can be made. This study identifies the need for baseline monitoring of cetaceans to further characterize the natural variability in skin gene expression in order to establish the value of skin transcriptomics as a tool for investigating exposures and the effects of stressors in free ranging animals.

## Supporting Information

S1 Table All samples included in this study, sorted by location. (XLS)

S2 Table Probes differentially expressed between males and females (annotated probes listed only). (XLS)

S3 Table Probes differentially expressed between winter and spring/summer (annotated probes listed only). (XLS)

S4 Table Gene Ontology categories enriched among gene probes differentially expressed in winter as compared to the warmer seasons. (XLS)

S5 Table Probes differentially expressed in Spring vs Summer 2010 (annotated probes listed only). (XLS)

S6 Table Probes differentially expressed between Summer 2010 and Summer 2011 (annotated probes listed only). (XLS)
